# Properties and usefulness of aggregates of synovial mesenchymal stem cells as a source for cartilage regeneration

**DOI:** 10.1186/ar3869

**Published:** 2012-06-07

**Authors:** Shiro Suzuki, Takeshi Muneta, Kunikazu Tsuji, Shizuko Ichinose, Hatsune Makino, Akihiro Umezawa, Ichiro Sekiya

**Affiliations:** 1 Section of Orthopedic Surgery, Graduate School, Tokyo Medical and Dental University, 1-5-45 Yushima, Bunkyo-ku, Tokyo 113-8519, Japan; 2 Global Center of Excellence Program, International Research Center for Molecular Science in Tooth and Bone Diseases, Tokyo Medical and Dental University, 1-5-45 Yushima, Bunkyo-ku, Tokyo 113-8519, Japan; 3 Instrumental Analysis Research Center, Tokyo Medical and Dental University, 1-5-45 Yushima, Bunkyo-ku, Tokyo 113-8519, Japan; 4 Department of Reproductive Biology, National Research Institute for Child Health and Development, 2-10-1 Okura, Setagaya-ku, Tokyo 157-8535, Japan; 5 Section of Cartilage Regeneration, Graduate School, Tokyo Medical and Dental University, 1-5-45 Yushima, Bunkyo-ku, Tokyo 113-8519, Japan

## Abstract

**Introduction:**

Transplantation of mesenchymal stem cells (MSCs) derived from synovium is a promising therapy for cartilage regeneration. For clinical application, improvement of handling operation, enhancement of chondrogenic potential, and increase of MSCs adhesion efficiency are needed to achieve a more successful cartilage regeneration with a limited number of MSCs without scaffold. The use of aggregated MSCs may be one of the solutions. Here, we investigated the handling, properties and effectiveness of aggregated MSCs for cartilage regeneration.

**Methods:**

Human and rabbit synovial MSCs were aggregated using the hanging drop technique. The gene expression changes after aggregation of synovial MSCs were analyzed by microarray and real time RT-PCR analyses. *In vitro *and *in vivo *chondrogenic potential of aggregates of synovial MSCs was examined.

**Results:**

Aggregates of MSCs cultured for three days became visible, approximately 1 mm in diameter and solid and durable by manipulation; most of the cells were viable. Microarray analysis revealed up-regulation of chondrogenesis-related, anti-inflammatory and anti-apoptotic genes in aggregates of MSCs. *In vitro *studies showed higher amounts of cartilage matrix synthesis in pellets derived from aggregates of MSCs compared to pellets derived from MSCs cultured in a monolayer. In *in vivo *studies in rabbits, aggregates of MSCs could adhere promptly on the osteochondral defects by surface tension, and stay without any loss. Transplantation of aggregates of MSCs at relatively low density achieved successful cartilage regeneration. Contrary to our expectation, transplantation of aggregates of MSCs at high density failed to regenerate cartilage due to cell death and nutrient deprivation of aggregates of MSCs.

**Conclusions:**

Aggregated synovial MSCs were a useful source for cartilage regeneration considering such factors as easy preparation, higher chondrogenic potential and efficient attachment.

## Introduction

Synovial mesenchymal stem cells (MSCs) are an attractive cell source for cartilage regeneration because of their high expansion and chondrogenic potentials [1-5]. We previously reported that more than 60% of synovial mesenchymal stem cells placed on osteochondral defects adhered to the defect within 10 minutes and promoted cartilage regeneration [6,7]. With this local adherent technique, we can transplant synovial MSCs without scaffold. One of the disadvantages in this method is that the cell component in the suspension is invisible to the naked eye.

One of the solutions for this problem is to make aggregates of synovial MSCs [8-10]. This could enable MSCs not only to be visible but also to be heavier. Consequently, aggregates of MSCs will sink faster in the suspension medium than dispersed MSCs. The use of aggregates of MSCs may help to avoid loss of MSCs from targeted cartilage defects and improve the procedures of transplantation of synovial MSCs. However, there are still concerns; properties of synovial MSCs will be altered when synovial MSCs are aggregated. We do not know whether aggregates of MSCs adhere on the cartilage defect as we expect it will, and the proper number of aggregates is unclear.

In this study, properties of aggregates of human synovial MSCs were analyzed from the standpoints of morphology, gene profile and *in vitro *chondrogenic potential. Also, the effect of transplantation of aggregates of synovial MSCs was investigated in a rabbit cartilage defect model in terms of aggregate number, cell behavior and influential factors in the *in vivo *chondrogenesis of aggregates of synovial MSCs. Finally, we demonstrated the usefulness of aggregates of synovial MSCs as a source for cartilage regeneration therapy.

## Materials and methods

### Isolation and culture of human synovial MSCs

This study was approved by an institutional review board of Tokyo Medical and Dental University (No.1030), and informed consent was obtained from all subjects. Human synovium was harvested from donors during anterior cruciate ligament reconstruction surgery for ligament injury and digested in a 3 mg/ml collagenase D solution (Roche Diagnostics, Mannheim, Germany) in α-minimal essential medium (αMEM) (Invitrogen, Carlsbad, CA, USA) at 37°C. After three hours, digested cells were filtered through a 70 μm nylon filter (Becton, Dickinson and Company, Franklin Lakes, NJ, USA), and the remaining tissues were discarded. The digested cells were plated in a 150 cm^2 ^culture dish (Nalge Nunc International, Rochester, NY, USA) in complete culture medium (CCM): αMEM containing 10% fetal bovine serum (FBS; Invitrogen), 100 units/ml penicillin (Invitrogen), 100 μg/ml streptomycin (Invitrogen), and 250 ng/ml amphotericin B (Invitrogen) and incubated at 37°C with 5% humidified CO_2_. The medium was changed to remove nonadherent cells one day later and cultured for 14 days as passage 0, then replated at 100 cells/cm^2 ^in a 150 cm^2 ^culture dish, cultured for 14 days and cryopreserved as passage 1. To expand the cells, a frozen vial of the cells was thawed, plated in 60 cm^2 ^culture dishes, and incubated for four days in the recovery plate. These cells were replated at 100 cells/cm^2 ^in a 150 cm^2 ^culture dish (passage 3), and cultured for an additional 14 days. These passage 3 cells were harvested and used in this study.

### Isolation and culture of rabbit synovial MSCs

This study was approved by the Animal Experimentation Committee of Tokyo Medical and Dental University (No.0120296A). Wild type skeletally mature Japanese White Rabbit and GFP transgenic rabbits [11,12] were anesthetized with an intramuscular injection of 25 mg/kg ketamine hydrochloride and with an intravenous injection of 45 mg/kg sodium pentobarbital and 150 μg/kg medetomidine hydrochloride. Synovium was harvested aseptically from knee joints of the rabbits, and digested in a 3 mg/ml collagenase type V in aMEM for three hours at 37°C. The digested cells were plated at 5 × 10^4 ^cells/cm^2 ^in a 150 cm^2 ^culture dish in CCM and incubated at 37°C with 5% humidified CO_2_. The medium was changed to remove nonadherent cells one day later and cultured for seven days as passage 0. The cells were then trypsinized, harvested and resuspended to be used for further assays. The cells that were transplanted in animals to be sacrificed at Day 0 and Day 14 were labeled for cell tracking by the fluorescent lipophilic tracer DiI (Molecular Probes, Eugene, OR, USA). For labeling, synovial MSCs were resuspended at 1 × 10^6 ^cells/ml in αMEM without FBS and a DiI was added at a final concentration of 5 μl/ml. After incubation for 20 minutes at 37°C with 5% humidified CO_2_, the cells were centrifuged at 450 *g *for 5 minutes and washed twice with phosphate-buffered saline (PBS) and the cells were then resuspended in CCM and cultured in hanging drops. We already reported that these cells had characteristics of MSCs [3,6,7,11].

### Preparation of aggregates of synovial MSCs

A total of 2.5 × 10^5 ^synovial MSCs were trypsinized, harvested and resuspended in 35 μl of CCM, plated on an inverted culture dish lid. The lid was inverted and placed on a culture dish containing PBS. The cells were cultured at 37°C with 5% humidified CO_2 _for three days in hanging drops.

### Histology of aggregates of human synovial MSCs

Aggregates of human synovial MSCs were fixed with 2.5% glutaraldehyde in 0.1 M PBS for two hours. The aggregates were washed overnight at 4°C in the same buffer and post-fixed with 1% OsO4 buffered with 0.1 M PBS for two hours. The aggregates were dehydrated in a graded series of ethanol and embedded in Epon 812. Semi-thin (1 μm) sections for light microscopy were collected on glass slides and stained for 30 seconds with toluidine blue.

### *In vitro *chondrogenic differentiation assay

A total of 2.5 × 10^5 ^human synovial MSCs cultured as a monolayer were pelleted by trypsinization and centrifugation. The pellets or aggregate of human synovial MSCs cultured for three days in hanging drops were cultured in 400 μl chondrogenic medium consisting of high-glucose Dulbecco's modified Eagle's medium (Invitrogen) supplemented with 1,000 ng/ml *BMP-7 *(Stryker Biotech, Boston, MA, USA), 10 ng/ml transforming growth factor-β3 (R&D Systems, Minneapolis, MN, USA), 100 nM dexamethasone (Sigma-Aldrich Corp., St. Louis, MO, USA), 50 μg/ml ascorbate-2-phosphate, 40 μg/ml proline, 100 μg/ml pyruvate, and 1:100 diluted ITS+Premix (6.25 μg/ml insulin, 6.25 μg/ml transferrin, 6.25 ng/ml selenious acid, 1.25 mg/ml bovine serum albumin, and 5.35 mg/ml linoleic acid; BD Biosciences Discovery Labware, Bedford, MA, USA). The medium was changed every 3 to 4 days for 21 days.

### Histology of pellets of human synovial MSCs

The pellets were embedded in paraffin, cut into 5-μm sections and stained with 1% Toluidine Blue. For immunohistochemistry, sections were treated with 0.4 mg/ml proteinase K (DAKO, Carpinteria, CA, USA) in Tris-HCl and normal horse serum after deparaffinization. Primary antibodies for type II collagen (Daiichi Fine Chemical, Toyama, Japan) and a secondary antibody of biotinylated horse anti-mouse IgG (Vector Laboratories, Burlingame, CA, USA) were employed. Immunostaining was detected with VECTASTAIN ABC reagent (Vector Laboratories) followed by 3,3'-diaminobenzidine staining.

### Real-time RT PCR analysis

Total RNA was extracted from human synovial MSCs in a monolayer culture, aggregates of human synovial MSCs cultured for 1, 2 and 3 days, and the pellets cultured for 7, 14 and 21 days using QIAzol (Qiagen, Hiden, Germany) and the RNeasy mini kit (Qiagen). cDNA was synthesized with oligo-dT primer from total RNA using the Transcriptor High Fidelity cDNA Synthesis kit (Roche Diagnostics) according to the manufacturer's protocol. Reverse transcription (RT) was performed by 30 minutes incubation at 55°C followed by 5 minutes incubation at 85°C. Real-time PCR was performed in a LightCycler 480 instrument (Roche Diagnostics). Primer sequences and TaqMan probes are listed in Table 1. After an initial denaturation step (95°C for 10 minutes), amplification was performed for 40 cycles (95°C for 15 seconds, 60°C for 60 seconds). Relative amounts of mRNA were calculated and standardized as previously described [13,14].

**Table 1:** Real time-RT PCR primer sequences

Primer name		Sequences	**Probe No**.
***β-actin***	forward	5'-ATTGGCAATGAGCGGTTC-3'	11
	reverse	5'-TGAAGGTAGTTTCGTGGATGC-3'	
***BMP2***	forward	5'-CGGACTGCGGTCTCCTAA-3'	49
	reverse	5'-GGAAGCAGCAACGCTAGAAG-3'	
***SOX5***	forward	5'-TCTGTCCCAGCAGCGTTAG-3'	41
	reverse	5'-TGACAGCATCATGGTCATTTAAG-3'	
***SOX6***	forward	5'-GCTTCTGGACTCAGCCCTTTA-3'	50
	reverse	5'-GGCCCTTTAGCCTTTGGTTA-3'	
***SOX9***	forward	5'-GTACCCGCACTTGCACAAC-3'	61
	reverse	5'-TCGCTCTCGTTCAGAAGTCTC-3'	
***TSG6***	forward	5'-CCAGATGACATCATCAGTACAGG-3'	78
	reverse	5'-CATTGCAACATATTTGATTTGGA-3'	
***STC1***	forward	5'-CCCAATCACTTCTCCAACAGA-3'	40
	reverse	5'-TGCTGACTGTGTCTTCATCACA-3'	
***COL2A1***	forward	5'-GTGTCAGGGCCAGGATGT-3'	75
	reverse	5'-TCCCAGTGTCACAGACACAGAT-3'	
***AGGRECAN***	forward	5'-CTGGAAGTCGTGGTGAAAGG-3'	21
	reverse	5'-TCGAGGGTGTAGCGTGTAGA-3'	

### DNA microarray analysis

Total RNA was extracted from human synovial MSCs in a monolayer culture, aggregates of human synovial MSCs cultured for three days. Human Genome U133 Plus 2.0 Array (GeneChip; Affymetrix, Santa Clara, CA, USA) containing the oligonucleotide probe set for more than 47,000 transcripts was used. The fluorescence intensity of each probe was quantified by using the GeneChip Analysis Suite 5.0 (Affymetrix). Gene expression data were normalized in Robust MultiChip Analysis (RMA). To analyze the data, we used hierarchical clustering using TIGR MultiExperiment Viewer (MeV) [15]. The microarray data have been deposited to the public database (GEO accession# GSE 31980).

### *In vivo *transplantation

Under anesthesia, the left knee joint was approached through a medial parapatellar incision, and the patella was dislocated laterally. Full-thickness osteochondral defects (5 mm × 5 mm wide, 1.5 mm deep) were created in the trochlear groove of the femur. A total of 5, 10, 20, 40 and 80 aggregates of autologous rabbit synovial MSCs (2.5 × 10^5 ^cells/aggregate) or 25 and 100 smaller aggregates of autologous rabbit synovial MSCs (1.0 × 10^5 ^cells/aggregate) suspended in PBS were transplanted to the defect. To trace the transplanted cells, DiI-labeled aggregates of autologous rabbit synovial MSCs and aggregates of allogenic synovial MSCs derived from GFP transgenic rabbit were transplanted to the defect. For the control group, the defect was left empty. All rabbits were returned to their cages after the operation and were allowed to move freely. Animals were sacrificed with an overdose of sodium pentobarbital at 1, 2, and 4 days and at 12 weeks after the operation (*n *= 5 at each time).

### Macroscopic examination

The cartilage defects were examined macroscopically for color, integrity and smoothness. Osteoarthritic changes and synovitis of the knee were also investigated. Digital images were taken using an Olympus MVX10 (Olympus, Tokyo, Japan).

### Histological examination and fluorescent microscopic examination

The dissected distal femurs were immediately fixed in a 4% paraformaldehyde (PFA) solution. The specimens were decalcified in 4% ethylenediamine tetraacetic acid solution, dehydrated with a gradient ethanol series and embedded in paraffin blocks. Sagittal sections 5 μm thick were obtained from the center of each defect and were stained with toluidine blue and Safranin O. For fluorescent microscopic examination and terminal deoxynucleotidyl transferase dUTP nick end labeling (TUNEL) staining, the fixed specimens were incubated at 4°C for three hours in 5%, 10%, 15% and 20% sucrose solution, respectively. After incubation, the fixed specimens were mounted on a holder. Then 30% optimal cutting temperature (OCT) (Sakura Finetek, Tokyo, Japan) in sucrose solution was added gently into the holder. The holder was frozen in hexan chilled by dry ice and stored at -80°C. Cryosections (10 μm) were prepared with an ultracut S microtome (Reichert, Wien, Austria) and a Microm HM560 cryostat.

### Histological score

Histological sections of the repaired tissue were analyzed using a grading system consisting of five categories (cell morphology, morphology, matrix staining, surface regularity, cartilage thickness and integration of donor with host), which were modified from the repaired cartilage score described by Wakitani and colleagues [16], so that overly thick, regenerated cartilage could not be overestimated [6]. The scoring was performed in a blinded manner by two observers and there was no significant interobserver difference. The ratio of the safranin-O positive area over the defect was evaluated. Zeiss AxioVison software (Carl Zeiss, Oberkochen, Germany) was used for measurement of defects and safranin-O positive areas.

### *In vitro *viability assay

Aggregates of rabbit synovial MSCs were plated at 1 or 40 aggregates/well in 96-well plates (Nunc) in CCM, and incubated at 37°C with 5% humidified CO_2 _for seven days without medium change. Aggregates were fixed in 4% PFA for TUNEL staining.

### TUNEL staining

For TUNEL staining, an apoptosis *in situ *detection kit (Wako Pure Chemical Industries, Ltd, Osaka, Japan) was used. The frozen semi-thin sections were incubated with terminal deoxynucleotidyl transferase for 10 minutes at 37°C in a moist chamber. The sections were washed with 0.1 M PBS for 15 minutes. Peroxidase-conjugated antibody was then applied to the specimens at 37°C for 10 minutes in a moist chamber. The sections were developed with 3,3-diaminobenizidine and counterstained with methyl green.

### Statistical analysis

Comparisons between two groups were analyzed using the Mann-Whitney U test. Comparisons between multi groups were analyzed using the Kruskal-Wallis test and the Steel test. A *P-*value of < 0.05 was considered statistically significant.

## Results

### Appearance of aggregates of human synovial MSCs

**Figure 1:**
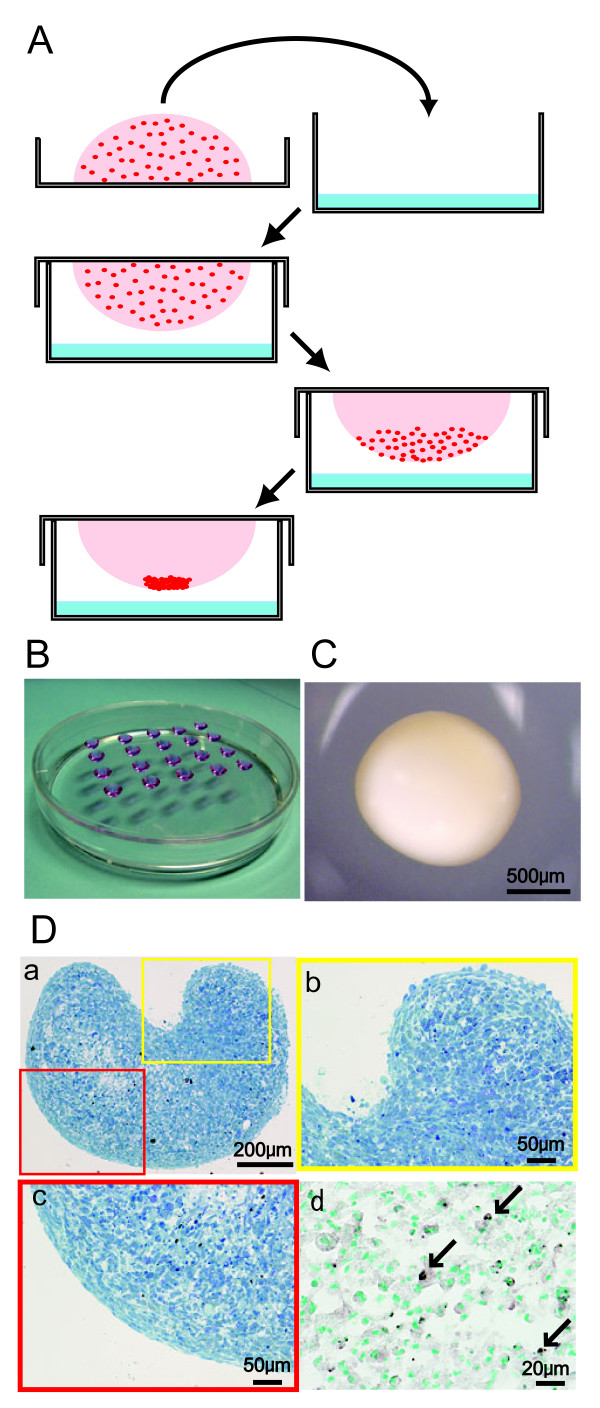
**Preparation and appearance of aggregates of human synovial MSCs**. **(A)**: Scheme of preparation of aggregates using hanging drop technique. **(B): **Drops hanging on the cover of 15 cm dish. **(C): **Macroscopic image of aggregate consisted of 250,000 MSCs, three days after cultured in hanging drop. **(D): **Sagittal sections of aggregates stained with toluidine blue (a, b, c) and TUNEL (d). TUNEL positive cells are indicated with arrows.

Human synovial MSCs were aggregated using the hanging drop technique (Figure 1A). Three days after being cultured in the drop (Figure 1B), the aggregate, consisting of 250,000 MSCs, became approximately 1 mm in diameter (Figure 1C). The aggregate was not easily broken by manipulation. Sagittal sections of the aggregates showed heart-shape as a whole (Figure 1Da). The superficial layer was composed of spindle cells parallel to the surface, whereas the deep layer was comprised of round cells both at top and bottom of the aggregate (Figure 1Db, c). Though cells positive for TUNEL staining were observed, the number was only approximately under 5% (Figure 1Dd).

### Transcriptome profile of aggregates of human synovial MSCs

**Figure 2:**
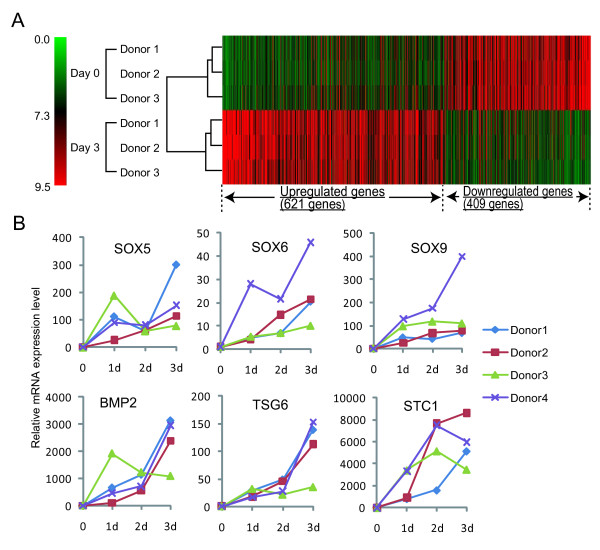
**Transcriptome changes after aggregation of human synovial MSCs**. **(A)**: Hierarchical clustering analysis for gene expression profile of aggregates of MSCs. The color code for the signal strength in the classification scheme is shown in the box left. High expression genes are indicated by shades of red and low expression genes are indicated by shades of green. **(B): **Expressions of chondrogenesis-related genes (*SOX5, SOX6, SOX9, BMP2*) and anti-inflammatory genes (*TSG-6, STC-1*) in aggregates of MSCs at Days 0 to 3 by real time RT-PCR analysis. The results are shown in four individual donors respectively.

To examine the sequential changes of gene expression profiles during aggregation of human synovial MSCs, microarray analyses were performed. The differences of gene profile between before and after aggregation exceeded those among donor variances (Figure 2A). The number of genes up-regulated more than five-fold was 621. The number of genes up-regulated more than 100-fold was 10, and these genes were related to hypoxia (integrin, alpha 2 (*ITGA2*), stanniocalcin 1 (*STC1*), chemokine (C-X-C motif) receptor 4 (*CXCR4*)), nutrient (*BMP2*, proprotein convertase subtilisin/kexin type 1 (*PCSK1*), secreted phosphoprotein 1 (*SPP1*), *ITGA2*, *STC1*), extracellular region (*MMP1*, *MMP3*), and cell adhesion (*SPP1*, *ITGA2*) (Table 2). The most up-regulated gene was *BMP2*, increased to 273 folds (Table 2). *STC1 *was also highly up-regulated in aggregates of synovial MSCs. The number of genes down-regulated less than one-fifth was 409, and the ontology for the genes was related to cell cycle. The microarray data are available at the public database (GEO accession# GSE 31980).

**Table 2:** The top 10 upregulated genes in aggregates of MSCs

**No**.	**Genebank No**.	Gene name	Symbol	fold change
1	AA583044	bone morphogenetic protein 2	***BMP2***	273
2	NM_002421	matrix metalloproteinase 1	***MMP1***	205
3	NM_000439	proprotein convertase subtilisin/kexin type 1	***PCSK1***	179
4	M86849	gap junction protein, beta 2	***GJB2***	170
5	M83248	secreted phosphoprotein 1 (osteopontin)	***SPP1***	156
6	L27624	tissue factor pathway inhibitor 2	***TFPI2***	137
7	NM_002422	matrix metalloproteinase 3	***MMP3***	136
8	N95414	integrin, alpha 2 (CD49B)	***ITGA2***	129
9	AW003173	stanniocalcin 1	***STC1***	124
10	AJ224869	chemokine (C-X-C motif) receptor 4	***CXCR4***	101

The top 10 genes which increased higher in aggregates of MSCs cultured in hanging drops for three days compared with MSCs in a monolayer culture.

Values are the means among three individual donors.

To further investigate gene expressions during aggregation of human synovial MSCs, real time RT-PCR analyses were additionally used for chondrogenesis-related genes (SRY (sex determining region Y)-box (*SOX)5, -6, -9*, and *BMP2*) and anti-inflammatory genes (TNFα inducible gene 6 (*TSG-6*), and *STC-1*) in four donors. In most cases, expressions for these genes increased sequentially (Figure 2B).

### *In vitro *chondrogenesis of aggregates of human synovial MSCs

**Figure 3:**
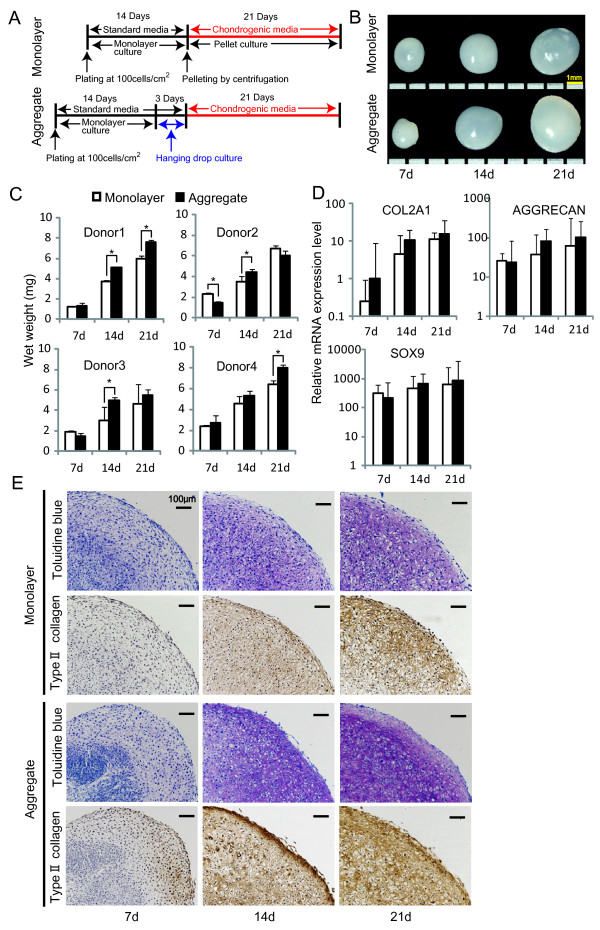
***In vitro *chondrogenic ability of human synovial MSCs after hanging drop culture (A): Scheme for the analyses**. **(B): **Macroscopic images of pellets derived from aggregates of MSCs and those of pellets derived from MSCs in a monolayer culture. **(C): **Wet weight in four individual donors. Values are the means with standard deviation (SD) (*P *< 0.05 by the Mann-Whitney U test). **(D): **Expressions of chondrogenesis-related genes by RT-PCR analyses. Values are the means with SD among four donors. The fold changes of *SOX9 *and *AGGRECAN *expression levels were shown when the gene expression levels at Day 0 were normalized as 1. The fold changes of *COL2A1 *expression levels were shown when the gene expression levels in MSCs in monolayer at Day 7 were normalized as 1 because *COL2A1 *expression level at Day 0 was undetectable. **(E): **Histological sections of pellets stained with toluidine blue and immunohistochemical analysis for type II collagen.

*In vitro *chondrogenic ability of human synovial MSCs after hanging drop culture was compared to that of MSCs after monolayer culture (Figure 3A). Aggregates of MSCs differentiated into chondrocytes as well (Figure 3B). The wet weight of pellets derived from MSCs after hanging drop culture was heavier than that of pellets derived from MSCs after monolayer culture in all four donors at 14 or 21 days (Figure 3C). Real time RT-PCR analysis showed higher expression levels of collagen *(COL)2A1*, aggrecan and *SOX9 *for pellets derived from MSCs after hanging drop culture compared to MSCs after monolayer culture at 14 and 21 days (Figure 3D). Cartilage extracellular matrix synthesis and accumulation of type II collagen were confirmed by histological analysis stained with toluidine blue and immunohistochemical analysis (Figure 3E).

### *In vivo *analysis for cartilage regeneration by transplantation of aggregates of synovial MSCs in rabbits

To examine whether transplantation of aggregates of synovial MSCs promotes cartilage regeneration, *in vivo *study was performed in rabbits. To further investigate the optimal number of aggregates consisting of 250,000 MSCs, 0 to 80 aggregates were transplanted into the defect.

**Figure 4:**
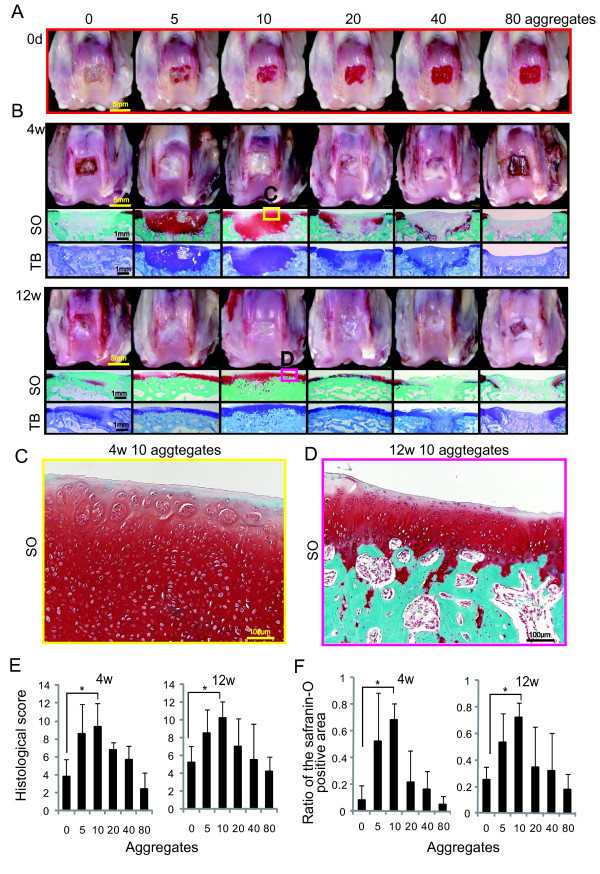
**Cartilage regeneration by transplantation of aggregates of synovial MSCs in rabbits**. **(A)**: Macroscopic observation of osteochondral defects one minute after transplantation of indicated number of aggregates of MSCs. The aggregate consisted of 250,000 MSCs, labeled with DiI for visualization. **(B): **Macroscopic and histological observation. For histologies, sagittal sections were stained with safranin-O (SO) and toluidine blue (TB). **(C, D): **Magnified histology of the indicated area. **(E): **Histological score. Values are the means with SD. (*n *= 5; *P *< 0.05 by the Kruskal-Wallis test and the Steel test). **(F): **Ratio of the safranin-O positive area to the defect area. Values are the means with SD. (*n *= 5; *P *< 0.05 by the Kruskal-Wallis test and the Steel test).

At 0 days, in the case of 40 and 80 transplanted aggregates, the osteochondral defects were filled with aggregates labeled with DiI macroscopically (Figure 4A).

At four weeks, in the case of 5 and 10 transplanted aggregates, the osteochondral defect was mostly covered with a thick cartilage matrix (Figure 4B, C). In the case of 20 and 40 transplanted aggregates, the defect was partially covered with cartilage matrix. In the case of 80 transplanted aggregates, the defect was filled with only fibrous tissue, which appeared to be similar to the control (Figure 4B).

At 12 weeks, in the case of 10 transplanted aggregates, the border between cartilage and bone moved up, and thickness of the regenerated cartilage became similar to the neighboring cartilage (Figure 4B, D). In the case of 5 and 20 transplanted aggregates, the bone defect was repaired, but the cartilage defect was filled partially with cartilage matrix. In the case of 40 and 80 transplanted aggregates, the osteochondral defect was poorly repaired, similar to the control (Figure 4B). Histological score was the best and the safranin-O positive area ratio was highest in the case of 10 transplanted aggregates both at 4 and 12 weeks (Figure 4E, F).

**Figure 5:**
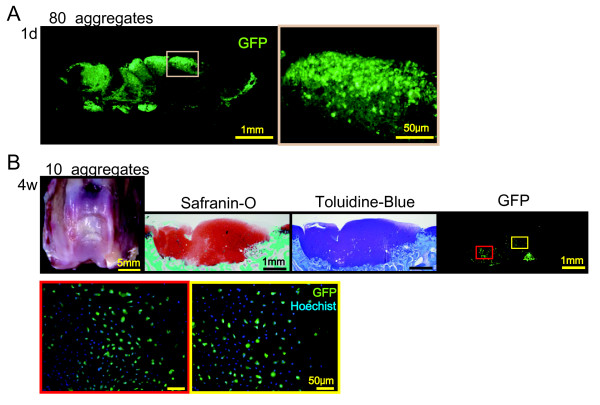
**Transplantation of 10 aggregates of synovial MSCs derived from a GFP rabbit**. **(A)**: Sagittal sections of osteochondral defect under fluorescence for GFP at one day. **(B): **Macroscopic and histological observation four weeks after transplantation. Nuclei were shown as blue in higher magnified pictures.

To trace MSCs, 10 aggregates of GFP positive MSCs were transplanted into the defect. At Day 1, no GFP positive aggregates could be observed in the knee joint except the defects with a fluorescent stereomicroscope. Histologically, aggregates changed their forms but have not fused yet (Figure 5A). At four weeks, the defect was filled with cartilage matrix and the GFP positive cells were still observed both at the bottom and the center of the regenerated cartilage (Figure 5B). Regenerated cartilage consisted of both GFP positive cells and GFP negative cells.

### Influences of cell number per aggregate and of aggregate number for transplantation

Cell number per aggregate as well as aggregate number may be a factor affecting properties of the aggregates. To answer this question, 25 or 100 aggregates, in which an aggregate consisted of 100,000 MSCs, were transplanted into the osteochondral defect.

**Figure 6:**
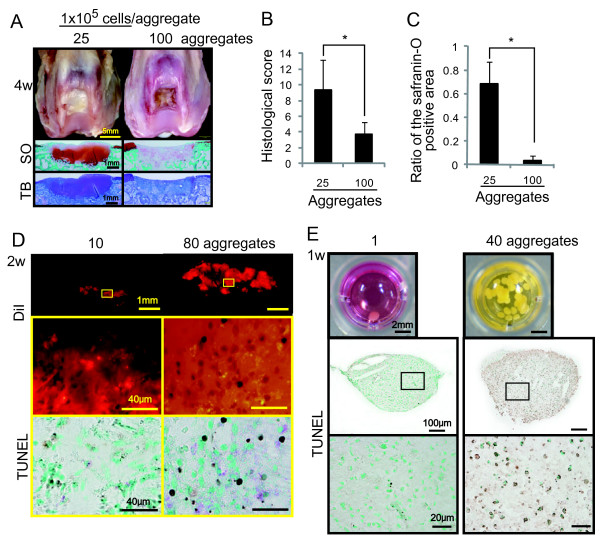
**Influences of cell number per an aggregate and of aggregate number for transplantation**. **(A)**: Macroscopic and histological observation four weeks after transplantation of 25 or 100 aggregates in which an aggregate consisted of 100,000 MSCs. **(B): **Histological score. Values are the means with SD (*n *= 4; *P *< 0.05 by the Mann-Whitney U test). **(C): **Ratio of the safranin-O positive area to the defect area. Values are the means with SD. (*n *= 4; *P *< 0.05 by the Mann-Whitney U test). **(D): **Histological observation two weeks after transplantation of 10 and 80 aggregates in which an aggregate consisted of 250,000 MSCs labeled with DiI. Sagittal sections under fluorescence and the serial sections stained with TUNEL were shown. **(E): ***In vitro *analyses of aggregates of rabbit synovial MSCs. One or 40 aggregates, in which an aggregate consisted of 250,000 MSCs, were cultured in a well of 96-well plates. Macroscopic images for the wells and sagittal sections of the aggregates stained with TUNEL were shown.

At four weeks, in the case of 25 transplanted aggregates, the defect was fully filled with cartilage matrix (Figure 6A), in which the result was different from the case of 20 or more aggregates, in which an aggregate consisted of 250,000 MSCs. In the case of 100 transplanted aggregates, the defect was filled with fibrous tissue, and the histological score was inferior and the safranin-O positive area ratio was smaller. (Figure 6B, C).

### Influences of aggregate number on viability of MSCs

To clarify why transplantation of aggregates over a certain number resulted in poor outcome, viability of cells was first examined by TUNEL staining. Compared to the case of 10 transplanted aggregates, much more TUNEL positive cells could be observed in the case of 80 transplanted aggregates (Figure 6D).

Another factor might be a nutrient deprivation and *in vitro *analyses using aggregates of rabbit synovial MSCs were performed. Seven days after 1 or 40 aggregates were cultured in a well of 96-well plates, the medium color changed to yellow in the case of 40 aggregates, while the color remained red in the case of only 1 aggregate (Figure 6E). TUNEL positive cells were much higher in the case of 40 aggregates than in the case of only 1 aggregate.

## Discussion

In this study, to form aggregates of synovial MSCs, the hanging drop technique was used [8-10]. This is a simple method; expensive or specific tools are not required. Three days after cultured in the drop, the aggregate, consisting of 250,000 MSCs, became approximately 1 mm in diameter, large enough to be visible and solid enough to aspirate with a pipette. Aggregates of MSCs sank faster in the suspension medium than dispersed MSCs and helped to avoid loss of MSCs from targeted cartilage defect. The use of aggregates was practically convenient for transplantation of MSCs.

In the previous report, the number of apoptotic or necrotic cells was greater in aggregates prepared with 100,000 or 250,000 human bone marrow MSCs, which was examined by flow cytometry, measuring propidium iodide uptake and annexin V labeling [10]. We examined the viability of aggregates of MSCs by TUNEL staining and confirmed that cells positive for TUNEL staining were observed; the number was small compared to the previous report. This difference may have been due to the difference of methods. Microarray analysis showed up-regulation of genes with ontology for regulation of cell death. The microarray data are available at the public database (GEO accession# GSE 31980). These results suggest that aggregation of 250,000 MSCs affect the viability of cells. However, we thought that aggregates of MSCs could be used as a source for cartilage regeneration because most cells which are cultured in drops for three days are viable.

Aggregation of synovial MSCs changed the gene expression profile dramatically without any special tools or chemical factors. This is possibly due to environmental changes, including cell-to-cell contact, hypoxic condition and low nutrient condition. Aggregation of human synovial MSCs increased expressions of several chondrogenesis-related genes and the most up-regulated gene was BMP2, which was also up-regulated in bone marrow MSCs [8,10].

In this study, we compared *in vitro *chondrogenesis potential of synovial MSCs after hanging drop culture with that of MSCs after monolayer culture. We used 1,000 ng/ml *BMP7 *for *in vitro *chondrogenic differentiation assay. We previously examined the dose effect of BMP6 between 0 to 500 ng/ml for *in vitro *chondrogenesis of bone marrow MSCs. Cartilage pellets increased in size along with the concentration of *BMP6*, and a maximal effect was at 500 ng/ml [17]. Our preliminary experiments showed that 1,000 ng/ml *BMP6 *induced larger cartilage pellets than 500 ng/ml *BMP6 *in bone marrow and synovial MSCs. We obtained similar results with *BMP7*. Real time RT-PCR analysis showed higher expression levels of *COL2A1*, aggrecan and *SOX9 *for pellets derived from MSC-aggregates after hanging drop culture compared to those of MSCs in a monolayer culture. Furthermore, the wet weight of pellets derived from MSC-aggregates after hanging drop culture was heavier than that of pellets derived MSCs in a monolayer culture. These indicate that chondrogenic potential increased in aggregates of MSCs after hanging drop culture.

In this study, we used an osteochondral defect model of rabbits, which have a higher, self-renewal capacity than bigger animals and humans. Therefore, the results obtained here should be critically evaluated. However, we prepared negative controls, which healed poorly at 4 and 12 weeks. We previously confirmed that the osteochondral defect created in the trochlear groove of the femur, similar to this study, was not repaired without any treatments 24 weeks after surgery [6]. These findings indicate that this rabbit model is useful to evaluate the effects of the treatments for cartilage regeneration.

For *in vivo *analysis of cartilage regeneration by transplantation of aggregates of synovial MSCs in rabbits, successful cartilage regeneration was observed in the cases of a relatively small number of transplanted aggregates of MSCs, and the worst results were observed when the highest number of aggregates of MSCs was transplanted. These results were not what we expected, because we previously reported that better cartilage regeneration was obtained when higher cell densities of MSCs were embedded in collagen gel [3].

Why were poor results obtained when more than a certain number of aggregates were transplanted? We listed three possible reasons. First, nutrition to maintain transplanted MSCs was depleted and the environment around transplanted MSCs worsened when too many aggregates were transplanted. As shown in Figure 6E, in the case of 40 aggregates that were cultured for seven days in a well of 96-well plates, medium color changed to yellow. This means that adjustment of pH could not be controlled. Second, TUNEL positive cells increased when too many aggregates were transplanted. The number of TUNEL positive cells was higher when too many aggregates were transplanted (Figure 6D) than before transplantation (Figure 1D) and after a suitable number of aggregates were transplanted (Figure 6D). Third, transplantation of too many aggregates prevented chondro-progenitor cells from moving to the osteochondral defect from bone marrow and from synovial fluid.

We confirmed that transplanted aggregates of synovial MSCs were directly differentiated into chondrocytes by transplanting MSCs derived from GFP transgenic rabbit. This result suggests that aggregates of synovial MSCs were involved in the reparative process. However, as shown in Figure 5B, in the case of aggregates of GFP positive MSCs being transplanted, regenerated cartilage consisted of both GFP positive cells and GFP negative cells. MSCs existed in synovial fluid [18] and these MSCs contributed to the repair of cartilage injury [6,19]. These results suggest that some host MSCs were also involved in the reparative process. In addition, host MSCs may have been involved in the anti-inflammatory process. In our rabbit osteochondral defect model, inflammation like a synovitis was not severe even in the control group. Therefore, we could not confirm the anti-inflammatory effect of MSCs. It would be interesting to investigate the anti-inflammatory effect of transplantation of aggregates of synovial MSCs and host MSCs in other arthritis models.

As previously reported, in bone marrow MSCs [10], aggregates of human synovial MSCs expressed anti-inflammatory genes *TSG6 *and *STC1*. *TSG6 *is secreted by synoviocytes, mononuclear cells and chondrocytes under inflammatory conditions and has an anti-inflammatory effect. Overexpression of *TSG6 *or administration of recombinant *TSG6 *inhibited inflammation and joint destruction in a murine collagen induced arthritis model [20-23]. *STC1 *is reported to have an anti-apoptotic effect as well as an anti-inflammatory effect [24,25]. However, their roles in joint homeostasis are unknown.

In this study, transplantation of low numbers of aggregates, in other words, low density of aggregates to the volume of the cartilage defect, showed better regeneration (Figures 4 and 6). This is favorable for clinical application. We have performed clinical trials of autologous human synovial MSCs transplantation for cartilage defects. In the experiences of 12 patients, approximately 50 million synovial MSCs at passage 0 were transplanted for approximately 280 mm^2 ^cartilage defects (unpublished data). In a rabbit model, we transplanted synovial MSC-aggregates into the osteochondral defects without any loss of cells, and 10 MSC-aggregates (2.5 × 10^6 ^cells) per 25 mm^2 ^defects were needed for better cartilage regeneration. According to these data, we can prepare a sufficient amount of human synovial MSCs at passage 0.

In this study, we did not use scaffolds for transplantation of aggregates of synovial MSCs. We were able to adhere aggregates of synovial MSCs on the osteochondral defect without scaffolds; however, the use of scaffolds or materials to improve survival of transplanted cells is attractive. One of the methods is the use of a fibrin glue, which has an effect of improving survival of transplanted cells [26]. In addition, cell transplantation of MSCs with a fibrin glue can probably be performed under arthroscopic surgery. Further studies are needed to improve cell transplantation procedures.

## Conclusion

Aggregated synovial MSCs were a useful source for cartilage regeneration considering such factors as easy preparation, higher chondrogenic potential and efficient attachment.

## Abbreviations

αMEM: α-minimal essential medium; *BMP*: bone morphogenetic protein; CCM: complete culture medium; *COL*: collagen; *CXCR4*: chemokine (C-X-C motif) receptor 4; EDTA: ethylenediaminetetraacetate; FBS: fetal bovine serum; GFP: green fluorescent protein; *GJB2*: gap junction protein, beta 2; *ITGA2*: integrin, alpha 2; MeV: MultiExperiment Viewer; MMP: matrix metalloproteinase; MSC: mesenchymal stem cell; OCT: optimal cutting temperature; PBS: phosphate-buffered saline; *PCSK1*: proprotein convertase subtilisin/kexin type 1; PFA: paraformaldehyde; RMA: Robust MultiChip Analysis; RT: reverse transcription; SD: standard deviation; SO: safranin-O; *SOX*: SRY (sex determining region Y)-box; *SPP1*: secreted phosphoprotein 1; *STC1*: stanniocalcin 1; TB: toluidine blue; *TFPI2*: tissue factor pathway inhibitor 2; *TNF*: tumor necrosis factor; *TSG6*: TNFα inducible gene 6; TUNEL: terminal deoxynucleotidyl transferase dUTP nick end labeling.

## Competing interests

The authors declare that they have no competing interests.

## Authors' contributions

SS participated in the design of the study, carried out the animal experiments, analyzed the results and drafted the manuscript. TM participated in the design of the study and provided the administrative and financial support. KT participated in the design of the study. SI helped with histological analysis. HM and AU carried out the microarray analysis and participated in the evaluation of the results. IS participated in the design of the study, provided the financial support and completed the final manuscript. All authors read and approved the final manuscript.
